# Supplementary material for: CAR Protects Females from Diet-Induced Steatosis and Associated Metabolic Disorders
Source: Cells. 2023 Sep 6;12(18):2218. doi: 10.3390/cells12182218 (PMC10527310; doi:10.3390/cells12182218)
Supplement: Supplementary file 1 [file cells-12-02218-s001.zip › cells-2590224-supplementary.pdf]

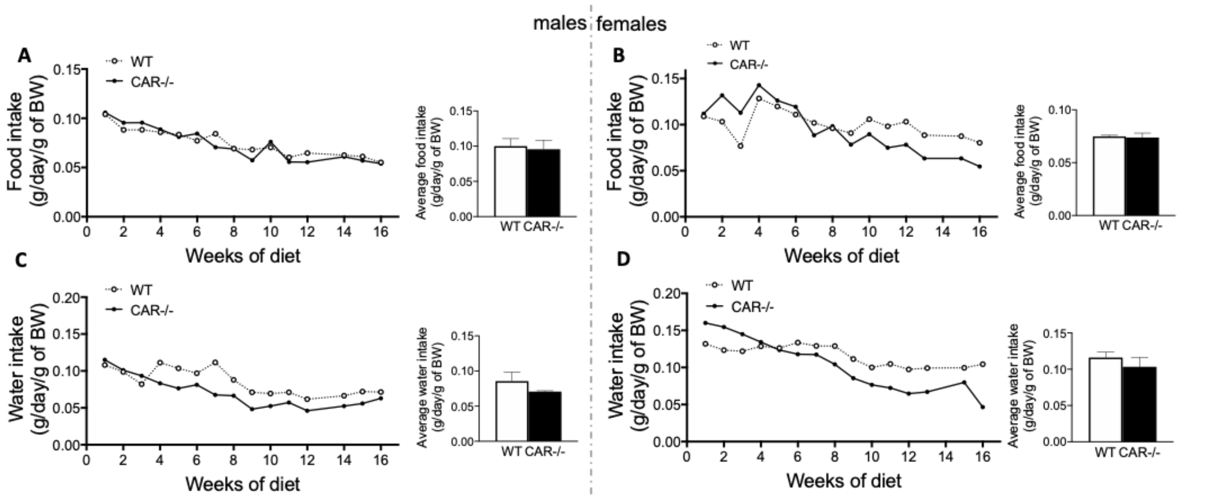

Supplementary Figure S1: CAR deletion does not affect food and water intake of mice. Food (A, B) and water (C, D) intake of males and females were measured weakly for each cage throughout the 16 weeks of HFD and averaged by day and by grams of mean body weight (BW) per cage (n=2 cages per group). An overall average of the 16 weeks was calculated.

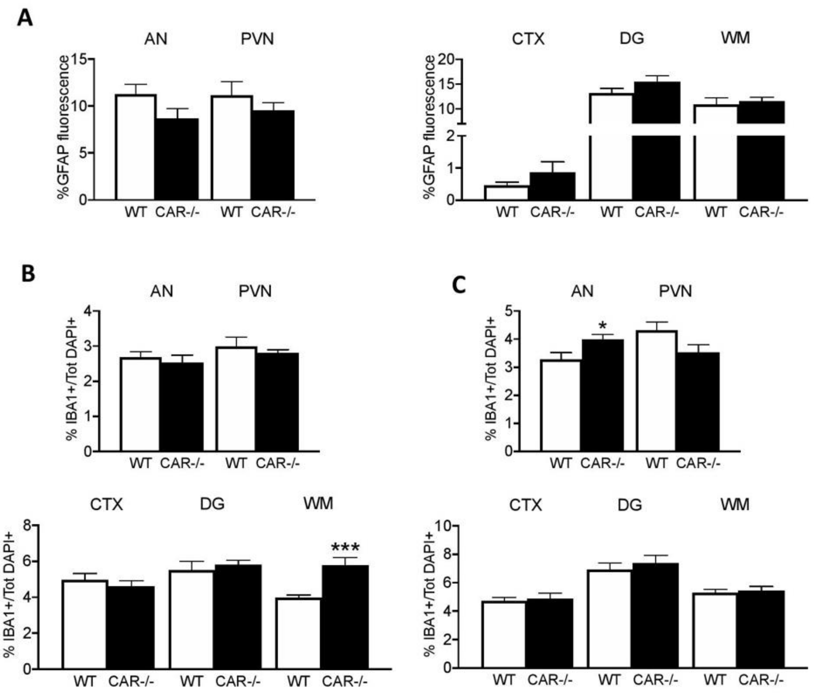

Supplementary Figure S2: CAR deletion induces dimorphic central adaptations. A: Quantification of GFAP expression in males, in the arcuate (AN) and paraventricular (PVN) nuclei of hypothalamus, Cortex (CTX), Dentate Gyrus (DG) and White Matter (WM). Histological slices were also stained for IBA1 expression, a microglial marker, in the same regions in males (B) and females (C).
`

Supplementary Table S1 : Precise criteria used in the NAS scoring system

Steatosis score				Inflammation score	Fibrosis score
Value	Area	Location	Morphology		
0	<5%	Zone 3		No foci	None
1	5-33%	Zone 1	Microvesicular	1 foci at 20x field	Zone 3 and/or perisinusoidal fibrosis
2	34-36%	Azonal		2 to 4 foci at 20x field	As grade 1 and portal fibrosis
3	>66%	Panacinar		>4 foci at 20x field	As grade 2 and bridging fibrosis
4	/	/	/	/	Cirrhosis

Supplementary Table S2. Oligonucleotide primers used for qPCR assays.

Gene	Forward 5′-3′	Reverse 3′-5′
Tnfα	TCCCCAAAGGGATGAGAAGTTC	GCGCTGGCTCAGCCACT
Il1β	GCCCATCCTCTGTGACTCAT	AGGCCACAGGTATTTTGTCG
Pdgfr1β	TGTGCAGTTGCCTTACGACT	CAGGTGGGGTCCAAGATGAC
Col1a1	AGCACGTCTGGTTTGGAGAG	GACATTAGGCGCAGGAAGGT
Tgfb1	GTCACTGGAGTTGTACGGCA	GGGGCTGATCCCGTTGATTT
Tgfbr1	GCATTGGCAAAGGTCGGTTT	TGCCTCTCGGAACCATGAAC
Acta2	AGCTACGAACTGCCTGACGG	CGTGGATGCCCGCTGAC

Supplementary Table S3: Primary/secondary antibodies used for immunohistochemistry

**Primary antibodies**	Host	Vendor	Reference	Dilution
Anti-IBA1	Anti-rabbit	Wako Laboratory Chemicals	019-19741	1/1000
Anti-GFAP	Anti-chicken	Abcam	Ab4674	1/300
DAPI	Vectashield : mountain medium for fluorescence with DAPI	Vector Laboratories	H-1200	[DAPI] = 1.5µg/ml
**Secondary antibodies**	Host	Vendor	Reference	Dilution
Anti-IBA1	Donkey anti-rabbit Alexa Fluor 488	Jackson ImmunoResearch	711-545-152	1/500
Anti-GFAP	Donkey anti-chicken Alexa Fluor Cy3	Jackson ImmunoResearch	703-165-155	1/500

Supplementary Table S4: Kegg pathway enrichment of cluster 3 and 7 of heat map

	Kegg pathways	P-value	Gene names
Cluster 3	Ribosome	1.134e-9	RPL30;RPL3;RPS5;MRPL15;RPL8;RPL9;RPL7;RPS26;RPS28;RPS27;RPS15A;RPS18;RPS3;RPL26;RPL15;RPS10;RPL28;RPS13
	Linoleic acid metabolism	2.431e-5	CYP2C68;CYP3A41A;CYP3A11;CYP3A44;CYP2C40;CYP2C70;CYP3A16
	Complement and coagulation cascades	1.515e-4	FGB;C3;F7;F10;SERPINF2;CFI;CFB;KNG1
	Chemical carcinogenesis	2.398e-4	ALDH3B2;CYP2C68;CYP3A41A;CYP3A11;CYP3A44;CYP2C40;CYP2C70;CYP3A16
	Steroid hormone biosynthesis	9.389e-4	CYP2C68;CYP3A41A;CYP3A11;CYP3A44;CYP2C40;CYP2C70;CYP3A16
	Retinol metabolism	0.001	CYP2C68;CYP3A41A;CYP3A11;CYP3A44;CYP2C40;CYP2C70;CYP3A16
	Adipocytokine signaling pathway	0.008	LEPR;AKT1;IRS2;PRKAB1;CAMKK2
Cluster 7	Drug metabolism	2.649e-8	UGT2B36;UGT2B35;MGST1;HPRT;FMO5;UGT1A6B;CES2B;CES2C;CES2E;GSTA3;CES1E;GSTA2;CMPK1;AOX1;GSTM6
	Metabolism of xenobiotics by cytochrome P450	1.239e-5	AKR7A5;UGT2B36;UGT2B35;GSTA3;GSTA2;MGST1;CYP1A1;UGT1A6B;GSTM6
	Ascorbate and aldarate metabolism	2.054e-5	UGDH;ALDH2;UGT2B36;UGT2B35;ALDH7A1;UGT1A6B
	Valine, leucine and isoleucine degradation	2.673e-5	BCKDHA;ECHS1;AUH;ALDH2;AGXT2;AOX1;ALDH7A1;HSD17B10
	Parkinson disease	3.183e-4	NDUFS5;NDUFA3;NDUFA10;NDUFB3;NDUFA2;UQCRC1;COX7A2;UQCR11;PARK7;PRKACA;GNAI1
	Alzheimer disease	4.645e-4	NDUFS5;NDUFA3;NDUFA10;NDUFB3;NDUFA2;UQCRC1;FAS;COX7A2;UQCR11;CAPN1;GAPDH;HSD17B10
	Thermogenesis	5.641e-4	COX19;NDUFA3;NDUFA10;NDUFB3;NDUFA2;UQCR11;COX7A2;ARID1B;ADCY6;NDUFS5;UQCRC1;PPARG;PRKACA;COA6
